# *catena*-Poly[[silver(I)-μ-bis­{2-[(*E*)-phenyl­diazen­yl]-1*H*-imidazol-1-yl}methane] trifluoro­methane­sulfonate]

**DOI:** 10.1107/S1600536811035951

**Published:** 2011-09-30

**Authors:** Tao Wang, Ji-Jun Xu, Chuan-Ming Jin

**Affiliations:** a Hubei Key Laboratory of Pollutant Analysis & Reuse Technology, College of Chemistry and Environmental Engineering, Hubei Normal University, Huangshi, 435002, People’s Republic of China

## Abstract

The title compound, {[Ag(C_19_H_16_N_8_)](CF_3_SO_3_)}_*n*_, is a coordin­ation polymer with cationic chain motif. The Ag^+^ cation is coordinated by two unsubstituted imidazolyl N atoms of two independent 2-paBIM ligands [2-paBIM is bis­{2-[(*E*)-phenyl­diazen­yl]-1*H*-imidazol-1-yl}methane]. The shortest Ag⋯Ag separation in a cationic chain is 8.841 (2) Å and the dihedral angle between two 2-phenyl­diazenyl-imidazole planes in the same ligand is 74.7 (3)°. Weak C—H⋯O interactions are seen in the crystal.

## Related literature

For background to metal-organic frameworks, see: Batten & Robson (1998 ▶); Burrows (2011 ▶); Leininger *et al.* (2000 ▶); Tanabe & Cohen (2011 ▶). For examples of supra­molecular arrangements using multidentate *N*-donor spacer ligands, see: Custelcean (2010 ▶); Pschirer *et al.* (2002 ▶). For structures of related ligands, see: Hamilton & Ziegler (2004 ▶); Jin *et al.* (2009 ▶).
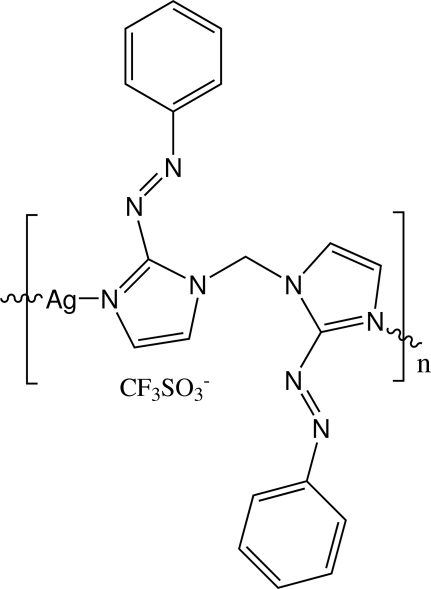


## Experimental

### 

#### Crystal data

[Ag(C_19_H_16_N_8_)](CF_3_SO_3_)
*M* *_r_* = 613.34
Orthorhombic, 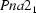

*a* = 16.0251 (19) Å
*b* = 8.455 (1) Å
*c* = 17.293 (2) Å
*V* = 2343.0 (5) Å^3^
*Z* = 4
Mo *K*α radiation
μ = 1.01 mm^−1^
*T* = 298 K
0.13 × 0.10 × 0.10 mm

#### Data collection

Bruker SMART CCD area-detector diffractometer
Absorption correction: multi-scan (*SADABS*; Sheldrick, 1996 ▶) *T*_min_ = 0.879, *T*_max_ = 0.905
11674 measured reflections
4823 independent reflections
4073 reflections with *I* > 2σ(*I*)
*R*_int_ = 0.057

#### Refinement

*R*[*F* ^2^ > 2σ(*F* ^2^)] = 0.065
*wR*(*F* ^2^) = 0.167
*S* = 1.13
4823 reflections
326 parameters
1 restraint
H-atom parameters constrained
Δρ_max_ = 1.08 e Å^−3^
Δρ_min_ = −0.54 e Å^−3^
Absolute structure: Flack (1983 ▶), 1807 Friedel pairs
Flack parameter: 0.54 (5)

### 

Data collection: *SMART* (Bruker, 2001 ▶); cell refinement: *SAINT-Plus* (Bruker, 2001 ▶); data reduction: *SAINT-Plus*; program(s) used to solve structure: *SHELXS97* (Sheldrick, 2008 ▶); program(s) used to refine structure: *SHELXL97* (Sheldrick, 2008 ▶); molecular graphics: *SHELXTL* (Sheldrick, 2008 ▶); software used to prepare material for publication: *SHELXTL*.

## Supplementary Material

Crystal structure: contains datablock(s) I, global. DOI: 10.1107/S1600536811035951/im2314sup1.cif

Structure factors: contains datablock(s) I. DOI: 10.1107/S1600536811035951/im2314Isup2.hkl

Additional supplementary materials:  crystallographic information; 3D view; checkCIF report

## Figures and Tables

**Table 1:** Selected bond lengths (Å)

Ag1—N3	2.152 (5)
Ag1—N6^i^	2.174 (6)

Symmetry code: (i) 
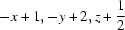
.

**Table 2:** Hydrogen-bond geometry (Å, °)

*D*—H⋯*A*	*D*—H	H⋯*A*	*D*⋯*A*	*D*—H⋯*A*
C10—H10*B*⋯O1^i^	0.97	2.50	3.285 (10)	138
C8—H8⋯O2^ii^	0.93	2.31	3.021 (10)	133

Symmetry codes: (i) 
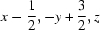
; (ii) 
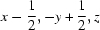
.

**Fig. 1.**
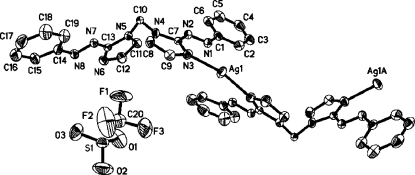
Structure of (I) showing the atom-numbering of one asymmetry unit. Displacement ellipsoids are drawn at the 30% probability level. H atoms have been omitted for clarity.

## supplementary crystallographic information

### Comment

The construction of functional metal-organic frameworks is of great interest due to their intriguing network topologies and their potential applications as microporous, magnetic or catalytically active species or in the fields of nonlinear optics, molecular separation, toxic materials adsorption and molecular sensors *etc.* (Batten & Robson, 1998; Burrows, 2011; Leininger *et al.*, 2000; Tanabe & Cohen, 2011). Such molecular architectures have been successfully designed and synthesized by judicious combination of a metal 'node' and an organic ligands as 'spacer'. The roles of counter anions and different solvent molecules are also of significant effect on supramolecular self-assembly. More recently, the molecular geometry and flexibility of multidentate N-donor spacer ligands play key roles in the field of molecular materials and supramolecular self-assemble crystal engineering. For example, 4, 4'-bipyridine, 1, 2-bis(4-pyridyl)ethane and *trans*-bis(4-pyridyl)ethene as ligands can form a lot of coordination polymers with different structural features (Custelcean, 2010; Pschirer *et al.*, 2002). The coordination polymer frameworks which were built by methylene C-bridged bipyridine, bitriazole and bisimidazole ligands have also been described widely (Hamilton & Ziegler, 2004; Jin *et al.*, 2009). Bis[2-((*E*)-phenyldiazenyl)-1*H*-imidazol-1-yl- methane (2-paBIM) is a flexibie V-shaped N-donor ligand containing azo groups which is built up by two methylene C-bridged substituted imidazole rings. The title compound, [Ag(2-paBIM)SO_3_CF_3_]_n_, (I), with a one-dimensional zigzag cationic chain structural motif was formed by the addition of a solution of 2-paBIM to AgSO_3_CF_3_.

Single crystal X-ray diffraction analysis reveals that complex (I) consists of one-dimensional cationic polymeric chains and uncoordinated CF_3_SO_3_^-^. The Ag^+^ ions are coordinated by two imidazolyl unsubstituted nitrogen atoms of independent 2-paBIM ligands, which act as bridges between silver(I) centers (Fig.1). The Ag^+^ ions show a coordination mode that is bent out of linearity with the bond angles of N—Ag—N being 153.9 (2)°. Ag—N bond lengths are 2.157 (5) Å and 2.170 (6) Å, respectively. Adjacent Ag······Ag distances in the same cationic chain are 8.841 (2) Å and the dihedral angle of the two 2-phenyldiazenyl-imidazole planes in the same ligand is 74.7 (3)°. Non-coordinated CF_3_SO_3_^-^ anions are filled in the voids of each zigzag cationic chain and show through the weak C—H······O hydrogen-bond interactions (Table 1).

### Experimental

An CH_3_CN solution (5 ml) of 2-paBIM (178 mg, 0.5 mmol) was slowly diffused into an aqueous solution (5 ml) of AgSO_3_CF_3_(128 mg, 0.5 mmol) in a test tube. Red crystals of [Ag(2-paBIM)SO_3_CF_3_]_n_ were formed at the interface of solvent in two weeks and were obtained in 62% yield.

### Refinement

The structure was refined as a racemic twin using TWIN and BASF keywords. H atoms were positioned geometrically at distances of 0.93 (CH), and 0.97 (CH_2_) from the parent C atoms. A riding model was used during the refinement process. The *U*_iso_ values were constrained to be 1.2*U*_eq_ of the corresponding carrier atom.

### Crystal data

**Table:**

[Ag(C_19_H_16_N_8_)](CF_3_SO_3_)	*F*(000) = 1224
*M**_r_* = 613.34	*D*_x_ = 1.739 Mg m^−^^3^
Orthorhombic, *P**n**a*2_1_	Mo *K*α radiation, λ = 0.71073 Å
Hall symbol: P 2c -2n	Cell parameters from 4016 reflections
*a* = 16.0251 (19) Å	θ = 2.4–24.4°
*b* = 8.455 (1) Å	µ = 1.01 mm^−^^1^
*c* = 17.293 (2) Å	*T* = 298 K
*V* = 2343.0 (5) Å^3^	Block, red
*Z* = 4	0.13 × 0.10 × 0.10 mm

### Data collection

**Table:**

Bruker SMART CCD area-detector diffractometer	4823 independent reflections
Radiation source: fine-focus sealed tube	4073 reflections with *I* > 2σ(*I*)
graphite	*R*_int_ = 0.057
phi and ω scans	θ_max_ = 28.3°, θ_min_ = 2.4°
Absorption correction: multi-scan (*SADABS*; Sheldrick, 1996)	*h* = −21→16
*T*_min_ = 0.879, *T*_max_ = 0.905	*k* = −11→11
11674 measured reflections	*l* = −23→19

### Refinement

**Table:**

Refinement on *F*^2^	Secondary atom site location: difference Fourier map
Least-squares matrix: full	Hydrogen site location: inferred from neighbouring sites
*R*[*F*^2^ > 2σ(*F*^2^)] = 0.065	H-atom parameters constrained
*wR*(*F*^2^) = 0.167	*w* = 1/[σ^2^(*F*_o_^2^) + (0.0908*P*)^2^] where *P* = (*F*_o_^2^ + 2*F*_c_^2^)/3
*S* = 1.13	(Δ/σ)_max_ = 0.023
4823 reflections	Δρ_max_ = 1.08 e Å^−^^3^
326 parameters	Δρ_min_ = −0.54 e Å^−^^3^
1 restraint	Absolute structure: Flack (1983), 1807 Friedel pairs
Primary atom site location: structure-invariant direct methods	Flack parameter: 0.54 (5)

### Special details

**Table:**

Geometry. All e.s.d.'s (except the e.s.d. in the dihedral angle between two l.s. planes) are estimated using the full covariance matrix. The cell e.s.d.'s are taken into account individually in the estimation of e.s.d.'s in distances, angles and torsion angles; correlations between e.s.d.'s in cell parameters are only used when they are defined by crystal symmetry. An approximate (isotropic) treatment of cell e.s.d.'s is used for estimating e.s.d.'s involving l.s. planes.
Refinement. Refinement of *F*^2^ against ALL reflections. The weighted *R*-factor *wR* and goodness of fit *S* are based on *F*^2^, conventional *R*-factors *R* are based on *F*, with *F* set to zero for negative *F*^2^. The threshold expression of *F*^2^ > σ(*F*^2^) is used only for calculating *R*-factors(gt) *etc*. and is not relevant to the choice of reflections for refinement. *R*-factors based on *F*^2^ are statistically about twice as large as those based on *F*, and *R*- factors based on ALL data will be even larger.

### Fractional atomic coordinates and isotropic or equivalent isotropic displacement parameters (Å^2^)

**Table:**

	*x*	*y*	*z*	*U*_iso_*/*U*_eq_	
Ag1	0.47527 (3)	0.90148 (7)	0.39948 (4)	0.0682 (2)	
C1	0.3518 (4)	1.2960 (8)	0.3721 (3)	0.0509 (14)	
C2	0.3877 (5)	1.3393 (13)	0.4422 (5)	0.078 (2)	
H2	0.4239	1.2714	0.4680	0.093*	
C3	0.3678 (5)	1.4883 (12)	0.4732 (5)	0.078 (3)	
H3	0.3912	1.5201	0.5199	0.094*	
C4	0.3152 (6)	1.5845 (10)	0.4357 (6)	0.075 (2)	
H4	0.3035	1.6836	0.4563	0.090*	
C5	0.2785 (6)	1.5408 (12)	0.3679 (5)	0.078 (2)	
H5	0.2411	1.6083	0.3434	0.093*	
C6	0.2974 (5)	1.3950 (8)	0.3356 (5)	0.0589 (16)	
H6	0.2729	1.3647	0.2892	0.071*	
C7	0.3657 (3)	0.9567 (8)	0.2569 (3)	0.0423 (12)	
C8	0.3670 (4)	0.7515 (7)	0.1808 (4)	0.0528 (15)	
H8	0.3584	0.6836	0.1392	0.063*	
C9	0.4113 (4)	0.7196 (8)	0.2445 (4)	0.0541 (15)	
H9	0.4383	0.6243	0.2542	0.065*	
C10	0.2915 (3)	0.9926 (9)	0.1299 (3)	0.0516 (14)	
H10A	0.2600	0.9216	0.0969	0.062*	
H10B	0.2523	1.0625	0.1556	0.062*	
C11	0.3752 (4)	1.2323 (9)	0.0999 (4)	0.0630 (17)	
H11	0.3593	1.2961	0.1412	0.076*	
C12	0.4303 (5)	1.2674 (9)	0.0438 (4)	0.0623 (17)	
H12	0.4594	1.3623	0.0405	0.075*	
C13	0.3883 (3)	1.0371 (8)	0.0180 (3)	0.0445 (13)	
C14	0.3903 (4)	0.7183 (8)	−0.1108 (4)	0.0544 (15)	
C15	0.4215 (5)	0.7007 (11)	−0.1837 (4)	0.0690 (19)	
H15	0.4534	0.7812	−0.2053	0.083*	
C16	0.4061 (7)	0.5638 (13)	−0.2260 (6)	0.088 (3)	
H16	0.4262	0.5518	−0.2760	0.106*	
C17	0.3599 (7)	0.4471 (14)	−0.1909 (7)	0.093 (3)	
H17	0.3498	0.3540	−0.2179	0.112*	
C18	0.3283 (7)	0.4624 (11)	−0.1177 (6)	0.090 (3)	
H18	0.2972	0.3812	−0.0957	0.108*	
C19	0.3433 (6)	0.6010 (9)	−0.0767 (5)	0.070 (2)	
H19	0.3219	0.6143	−0.0271	0.084*	
C20	0.5958 (6)	−0.0608 (11)	0.1501 (5)	0.075 (2)	
F1	0.5243 (3)	0.0059 (14)	0.1521 (6)	0.152 (4)	
F2	0.5931 (9)	−0.2044 (11)	0.1290 (6)	0.199 (6)	
F3	0.6175 (6)	−0.0668 (10)	0.2239 (4)	0.131 (3)	
N1	0.3750 (3)	1.1435 (7)	0.3445 (3)	0.0501 (11)	
N2	0.3443 (3)	1.1075 (6)	0.2807 (3)	0.0462 (11)	
N3	0.4113 (3)	0.8465 (6)	0.2935 (3)	0.0484 (11)	
N4	0.3370 (3)	0.9007 (6)	0.1878 (3)	0.0450 (12)	
N5	0.3475 (3)	1.0842 (6)	0.0837 (3)	0.0427 (11)	
N6	0.4379 (4)	1.1474 (7)	−0.0069 (3)	0.0520 (12)	
N7	0.3705 (3)	0.8891 (6)	−0.0124 (3)	0.0465 (12)	
N8	0.4090 (3)	0.8640 (7)	−0.0747 (3)	0.0508 (12)	
O1	0.6671 (6)	0.1988 (10)	0.1246 (6)	0.127 (3)	
O2	0.7448 (5)	−0.0336 (14)	0.1010 (6)	0.149 (4)	
O3	0.6378 (5)	0.0472 (10)	0.0180 (4)	0.104 (2)	
S1	0.67017 (10)	0.0515 (2)	0.09234 (12)	0.0592 (4)	

### Atomic displacement parameters (Å^2^)

**Table:**

	*U*^11^	*U*^22^	*U*^33^	*U*^12^	*U*^13^	*U*^23^
Ag1	0.0678 (3)	0.0891 (4)	0.0477 (3)	−0.0057 (2)	−0.0201 (2)	0.0008 (3)
C1	0.047 (3)	0.059 (4)	0.047 (3)	−0.014 (3)	0.013 (2)	−0.013 (3)
C2	0.053 (4)	0.117 (7)	0.064 (5)	−0.010 (4)	0.000 (3)	−0.016 (5)
C3	0.066 (4)	0.104 (7)	0.064 (5)	−0.033 (5)	0.016 (4)	−0.048 (5)
C4	0.079 (6)	0.069 (5)	0.077 (6)	−0.016 (4)	0.024 (5)	−0.027 (4)
C5	0.082 (5)	0.077 (5)	0.074 (5)	0.002 (4)	0.024 (4)	−0.006 (4)
C6	0.063 (4)	0.056 (4)	0.058 (4)	−0.004 (3)	0.012 (3)	−0.006 (3)
C7	0.031 (2)	0.065 (4)	0.032 (3)	−0.006 (2)	0.0032 (19)	0.005 (2)
C8	0.070 (4)	0.045 (3)	0.044 (3)	−0.010 (3)	−0.001 (3)	−0.011 (3)
C9	0.059 (4)	0.044 (3)	0.059 (4)	−0.001 (3)	0.001 (3)	0.002 (3)
C10	0.033 (2)	0.092 (5)	0.030 (3)	0.000 (3)	0.001 (2)	−0.011 (3)
C11	0.058 (4)	0.080 (5)	0.051 (4)	0.010 (3)	0.006 (3)	−0.004 (3)
C12	0.071 (4)	0.058 (4)	0.058 (4)	−0.002 (3)	0.007 (3)	0.006 (3)
C13	0.032 (2)	0.069 (4)	0.033 (3)	0.004 (2)	−0.004 (2)	0.001 (2)
C14	0.057 (3)	0.065 (4)	0.041 (3)	0.013 (3)	−0.007 (3)	−0.006 (3)
C15	0.069 (4)	0.081 (5)	0.056 (4)	0.019 (4)	0.005 (3)	−0.015 (4)
C16	0.084 (6)	0.109 (8)	0.070 (6)	0.020 (5)	−0.004 (5)	−0.017 (5)
C17	0.098 (7)	0.091 (7)	0.090 (7)	0.017 (6)	−0.030 (6)	−0.039 (6)
C18	0.117 (7)	0.063 (5)	0.090 (8)	−0.007 (5)	0.000 (6)	−0.005 (5)
C19	0.085 (5)	0.066 (5)	0.059 (4)	−0.002 (4)	−0.001 (4)	0.000 (3)
C20	0.076 (5)	0.073 (5)	0.076 (6)	−0.010 (4)	0.007 (4)	0.029 (4)
F1	0.051 (3)	0.237 (9)	0.169 (8)	−0.006 (4)	0.022 (3)	0.111 (8)
F2	0.347 (16)	0.110 (5)	0.141 (8)	−0.123 (8)	0.069 (9)	−0.022 (5)
F3	0.159 (7)	0.167 (7)	0.065 (4)	0.015 (5)	0.009 (4)	0.049 (4)
N1	0.050 (3)	0.064 (3)	0.037 (2)	−0.006 (2)	−0.001 (2)	−0.002 (2)
N2	0.043 (2)	0.061 (3)	0.035 (2)	−0.003 (2)	0.0013 (19)	−0.003 (2)
N3	0.050 (3)	0.051 (3)	0.044 (3)	−0.005 (2)	−0.006 (2)	0.006 (2)
N4	0.051 (3)	0.051 (3)	0.033 (2)	−0.008 (2)	0.002 (2)	−0.0095 (19)
N5	0.039 (2)	0.054 (3)	0.035 (2)	0.0043 (18)	−0.0010 (19)	0.001 (2)
N6	0.049 (3)	0.061 (3)	0.047 (3)	0.008 (3)	0.006 (2)	0.008 (3)
N7	0.044 (2)	0.062 (3)	0.034 (2)	0.006 (2)	0.0006 (18)	0.000 (2)
N8	0.051 (3)	0.064 (3)	0.038 (2)	0.009 (2)	0.0003 (19)	0.000 (2)
O1	0.154 (8)	0.089 (5)	0.138 (8)	−0.037 (5)	0.043 (6)	−0.017 (5)
O2	0.076 (4)	0.222 (9)	0.148 (8)	0.060 (6)	0.018 (5)	0.058 (8)
O3	0.120 (6)	0.134 (6)	0.060 (4)	−0.030 (5)	−0.012 (4)	0.037 (4)
S1	0.0443 (7)	0.0712 (10)	0.0622 (10)	−0.0010 (7)	0.0022 (7)	0.0131 (9)

### Geometric parameters (Å, °)

**Table:**

Ag1—N3	2.152 (5)	C11—H11	0.9300
Ag1—N6^i^	2.174 (6)	C12—N6	1.347 (10)
C1—C6	1.364 (11)	C12—H12	0.9300
C1—C2	1.390 (10)	C13—N6	1.298 (9)
C1—N1	1.424 (9)	C13—N5	1.371 (7)
C2—C3	1.406 (14)	C13—N7	1.387 (8)
C2—H2	0.9300	C14—C15	1.364 (10)
C3—C4	1.339 (14)	C14—C19	1.378 (11)
C3—H3	0.9300	C14—N8	1.413 (8)
C4—C5	1.363 (13)	C15—C16	1.391 (13)
C4—H4	0.9300	C15—H15	0.9300
C5—C6	1.386 (11)	C16—C17	1.374 (16)
C5—H5	0.9300	C16—H16	0.9300
C6—H6	0.9300	C17—C18	1.369 (16)
C7—N3	1.342 (8)	C17—H17	0.9300
C7—N4	1.365 (8)	C18—C19	1.390 (12)
C7—N2	1.383 (8)	C18—H18	0.9300
C8—C9	1.338 (10)	C19—H19	0.9300
C8—N4	1.355 (8)	C20—F2	1.268 (13)
C8—H8	0.9300	C20—F1	1.278 (11)
C9—N3	1.366 (9)	C20—F3	1.322 (11)
C9—H9	0.9300	C20—S1	1.822 (8)
C10—N5	1.429 (8)	N1—N2	1.246 (7)
C10—N4	1.463 (9)	N6—Ag1^ii^	2.174 (6)
C10—H10A	0.9700	N7—N8	1.259 (7)
C10—H10B	0.9700	O1—S1	1.365 (9)
C11—C12	1.345 (10)	O2—S1	1.404 (7)
C11—N5	1.357 (9)	O3—S1	1.388 (7)
			
N3—Ag1—N6^i^	153.9 (2)	C19—C14—N8	123.7 (6)
C6—C1—C2	120.4 (7)	C14—C15—C16	120.8 (9)
C6—C1—N1	124.6 (6)	C14—C15—H15	119.6
C2—C1—N1	115.0 (7)	C16—C15—H15	119.6
C1—C2—C3	118.3 (9)	C17—C16—C15	117.5 (9)
C1—C2—H2	120.8	C17—C16—H16	121.3
C3—C2—H2	120.8	C15—C16—H16	121.3
C4—C3—C2	120.2 (7)	C16—C17—C18	122.6 (9)
C4—C3—H3	119.9	C16—C17—H17	118.7
C2—C3—H3	119.9	C18—C17—H17	118.7
C3—C4—C5	121.6 (8)	C17—C18—C19	119.2 (10)
C3—C4—H4	119.2	C17—C18—H18	120.4
C5—C4—H4	119.2	C19—C18—H18	120.4
C4—C5—C6	119.5 (10)	C14—C19—C18	118.9 (8)
C4—C5—H5	120.2	C14—C19—H19	120.6
C6—C5—H5	120.2	C18—C19—H19	120.6
C1—C6—C5	120.0 (8)	F2—C20—F1	113.5 (11)
C1—C6—H6	120.0	F2—C20—F3	104.5 (10)
C5—C6—H6	120.0	F1—C20—F3	103.1 (9)
N3—C7—N4	110.8 (6)	F2—C20—S1	111.3 (8)
N3—C7—N2	129.4 (6)	F1—C20—S1	111.7 (6)
N4—C7—N2	119.8 (5)	F3—C20—S1	112.2 (7)
C9—C8—N4	107.5 (6)	N2—N1—C1	114.6 (6)
C9—C8—H8	126.2	N1—N2—C7	113.0 (5)
N4—C8—H8	126.2	C7—N3—C9	104.6 (5)
C8—C9—N3	110.7 (6)	C7—N3—Ag1	120.7 (4)
C8—C9—H9	124.7	C9—N3—Ag1	134.2 (4)
N3—C9—H9	124.7	C8—N4—C7	106.4 (6)
N5—C10—N4	110.9 (4)	C8—N4—C10	127.6 (5)
N5—C10—H10A	109.5	C7—N4—C10	125.6 (5)
N4—C10—H10A	109.5	C11—N5—C13	106.4 (5)
N5—C10—H10B	109.5	C11—N5—C10	126.1 (5)
N4—C10—H10B	109.5	C13—N5—C10	127.3 (5)
H10A—C10—H10B	108.0	C13—N6—C12	105.6 (6)
C12—C11—N5	105.6 (6)	C13—N6—Ag1^ii^	120.2 (5)
C12—C11—H11	127.2	C12—N6—Ag1^ii^	133.3 (5)
N5—C11—H11	127.2	N8—N7—C13	112.0 (5)
C11—C12—N6	111.3 (7)	N7—N8—C14	114.9 (6)
C11—C12—H12	124.4	O1—S1—O3	112.9 (6)
N6—C12—H12	124.4	O1—S1—O2	117.1 (7)
N6—C13—N5	111.0 (6)	O3—S1—O2	113.8 (6)
N6—C13—N7	130.4 (6)	O1—S1—C20	103.1 (5)
N5—C13—N7	118.5 (5)	O3—S1—C20	104.5 (5)
C15—C14—C19	121.1 (7)	O2—S1—C20	103.4 (5)
C15—C14—N8	115.2 (7)		
			
C6—C1—C2—C3	−1.4 (11)	N2—C7—N4—C8	−179.3 (5)
N1—C1—C2—C3	179.9 (6)	N3—C7—N4—C10	−174.5 (5)
C1—C2—C3—C4	0.3 (12)	N2—C7—N4—C10	7.2 (8)
C2—C3—C4—C5	1.3 (12)	N5—C10—N4—C8	−91.0 (8)
C3—C4—C5—C6	−1.7 (12)	N5—C10—N4—C7	81.0 (7)
C2—C1—C6—C5	1.0 (10)	C12—C11—N5—C13	0.3 (7)
N1—C1—C6—C5	179.5 (6)	C12—C11—N5—C10	176.1 (6)
C4—C5—C6—C1	0.6 (11)	N6—C13—N5—C11	−0.8 (7)
N4—C8—C9—N3	−0.3 (8)	N7—C13—N5—C11	−179.1 (5)
N5—C11—C12—N6	0.2 (9)	N6—C13—N5—C10	−176.5 (5)
C19—C14—C15—C16	0.5 (12)	N7—C13—N5—C10	5.2 (9)
N8—C14—C15—C16	−178.8 (7)	N4—C10—N5—C11	−88.1 (7)
C14—C15—C16—C17	−1.2 (13)	N4—C10—N5—C13	86.8 (7)
C15—C16—C17—C18	1.1 (15)	N5—C13—N6—C12	0.9 (7)
C16—C17—C18—C19	−0.1 (16)	N7—C13—N6—C12	178.9 (6)
C15—C14—C19—C18	0.5 (12)	N5—C13—N6—Ag1^ii^	171.3 (4)
N8—C14—C19—C18	179.7 (8)	N7—C13—N6—Ag1^ii^	−10.6 (9)
C17—C18—C19—C14	−0.6 (14)	C11—C12—N6—C13	−0.7 (8)
C6—C1—N1—N2	3.2 (9)	C11—C12—N6—Ag1^ii^	−169.3 (5)
C2—C1—N1—N2	−178.2 (6)	N6—C13—N7—N8	−1.3 (9)
C1—N1—N2—C7	−177.8 (5)	N5—C13—N7—N8	176.7 (5)
N3—C7—N2—N1	2.4 (8)	C13—N7—N8—C14	−176.8 (5)
N4—C7—N2—N1	−179.7 (5)	C15—C14—N8—N7	170.3 (6)
N4—C7—N3—C9	0.8 (6)	C19—C14—N8—N7	−9.0 (9)
N2—C7—N3—C9	178.9 (6)	F2—C20—S1—O1	−177.9 (10)
N4—C7—N3—Ag1	174.6 (4)	F1—C20—S1—O1	54.1 (10)
N2—C7—N3—Ag1	−7.3 (8)	F3—C20—S1—O1	−61.1 (9)
C8—C9—N3—C7	−0.3 (7)	F2—C20—S1—O3	63.9 (10)
C8—C9—N3—Ag1	−172.8 (5)	F1—C20—S1—O3	−64.1 (10)
N6^i^—Ag1—N3—C7	−170.1 (5)	F3—C20—S1—O3	−179.4 (8)
N6^i^—Ag1—N3—C9	1.5 (9)	F2—C20—S1—O2	−55.5 (11)
C9—C8—N4—C7	0.8 (7)	F1—C20—S1—O2	176.5 (10)
C9—C8—N4—C10	174.1 (6)	F3—C20—S1—O2	61.3 (10)
N3—C7—N4—C8	−1.0 (7)		

Symmetry codes: (i) −*x*+1, −*y*+2, *z*+1/2; (ii) −*x*+1, −*y*+2, *z*−1/2.

### Hydrogen-bond geometry (Å, °)

**Table:**

*D*—H···*A*	*D*—H	H···*A*	*D*···*A*	*D*—H···*A*
C10—H10B···O1^iii^	0.97	2.50	3.285 (10)	138.
C8—H8···O2^iv^	0.93	2.31	3.021 (10)	133.

Symmetry codes: (iii) *x*−1/2, −*y*+3/2, *z*; (iv) *x*−1/2, −*y*+1/2, *z*.
